# The complete mitochondrial genome of the widely cultivated edible fungus *Lentinula edodes*

**DOI:** 10.1080/23802359.2016.1275839

**Published:** 2017-01-11

**Authors:** Ruiheng Yang, Yan Li, Xiaoxia Song, Lihua Tang, Chuanhua Li, Qi Tan, Dapeng Bao

**Affiliations:** National Engineering Research Center of Edible Fungal Ministry of Science and Technology (MOST), Key Laboratory of Edible Fungi Resources and Utilization (South), Key Laboratory of Agricultural Genetics and Breeding of Shanghai, Institute of Edible Fungi Shanghai Academy of Agricultural Sciences, Shanghai, China

**Keywords:** *Lentinula edodes*, agaricales, omphalotaceae, mitogenome

## Abstract

The complete mitochondrial genome of the widely cultivated edible fungus *Lentinula edodes* was determined using the next-generation sequencing technology. The circular molecule is 116,819 bp in length with a GC content of 30.75%. Conserved genes including 13 putative protein-coding genes and 24 tRNAs were located on the same strand. We detected 14 introns invading 4 genes, including cob, cox1, nad1, and nad5. The phylogenetic analysis confirmed that *L. edodes* was a number of Agaricales. This mitochondrial genome may open new avenues for understanding the phylogeny and evolution of Omphalotaceae and Agaricales.

The mushroom *Lentinula edodes* (Berk.) Pegler, named as Xianggu in China, is one of the most important and popular edible mushrooms all over the world, especially in East Asia (Chang 1999). It was reported that the cultivation of *L. edodes* was originated from China, and spread to Japan and other Far East Asian countries (Chang 1999). Several studies have focused on genetic diversities and relationships between strains originated from different regions (Hibbett & Donoghue 1996; Gong et al. 2016, Li et al. 2008). Here, a complete mitogenome of wild *L. edodes* in China was reported, which might provide new insights into genetic structure and differentiation of this fungus.

The monokaryon strain of wild *L. edodes* was isolated from dikaryon strain collected from Guizhou Province (China) using protoplast isolation method published previously (Chang et al. 1985). The living culture was deposited at the Guangdong Microbiology Culture Center (GDMCC 5.566). The whole-genome sequencing was conducted using Hiseq sequencing. A total of 7000 M high-quality reads was generated from 350 insert-size library and assembled using A5-miseq 2.0 (New South Wales, Australia, Coil et al. 2014). All the assembled contigs were mapped to the database of fungal mitogenomes to extract contigs belonging to the mitogenome of *L. edodes*. The completed mitogenome was annotated using MFannot (http://megasun.bch.umontreal.ca/cgi-bin/mfannot/mfannotInterface.pl). The phylogenetic analysis of 18 other species belonged to Agaricomycotina conducted based on the neighbour-joining method using the software MEGA 7.0 (Tokyo, Japan, Kumar et al. 2016) (Figure 1).

**Figure 1.**
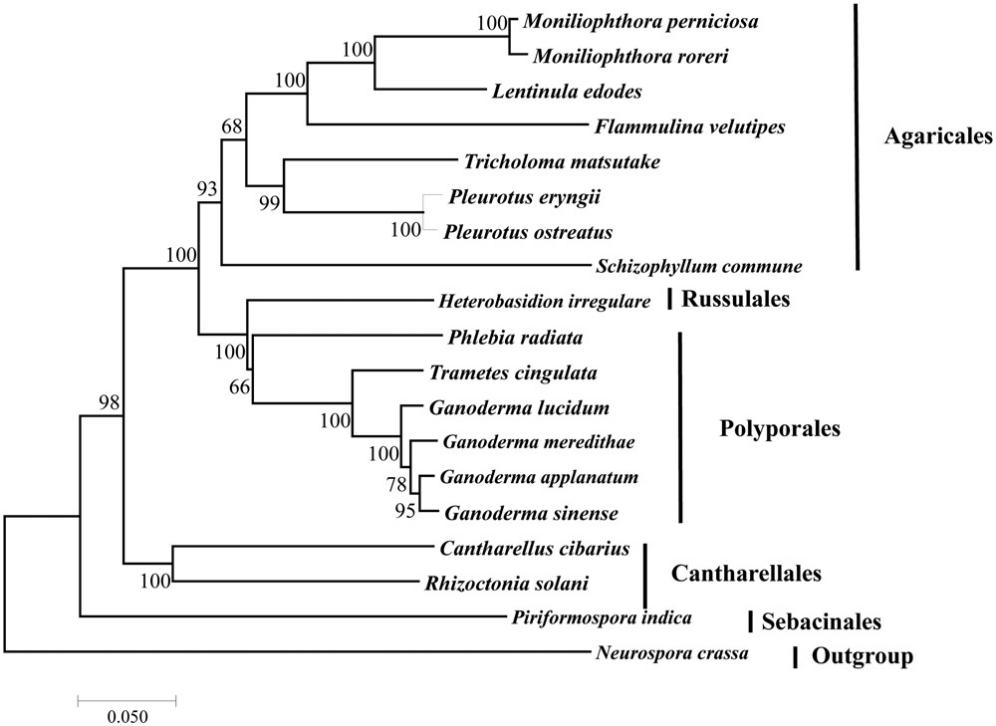
Phylogenetic analysis of 18 species of Agaricomycotina (including *L. edodes*) conducted based on the NJ method implemented in MEGA 7.0 (Kumar et al. 2016). A total of 11 amino acid sequences were used, including atp8, atp9, cob, cox1, cox2, cox3, nad1, nad3, nad4L, nad5, and nad6. The concatenated sequences were aligned using Clustal (Thompson et al. 2010). All the sequences could be currently available in the GenBank database: *Cantharellus cibarius* (NC_020368), *Flammulina velutipes* (NC_021373), *Ganoderma applanatum* (NC_027188), *Ganoderma lucidum* (NC_021750), *Ganoderma meredithae* (NC_026782), *Ganoderma sinense* (NC_022933), *Heterobasidion irregulare* (NC_024555), *Moniliophthora perniciosa* (NC_005927), *Moniliophthora roreri* (NC_015400), *Phlebia radiata* (NC_020148), *Pleurotus eryngii* (KX827267), *Pleurotus ostreatus* (NC_009905), *Rhizoctonia solani* (HF546977), *Schizophyllum commune* (NC_003049), *Serendipita indica* (FQ859090), *Trametes cingulata* (NC_013933), and *Tricholoma matsutake* (NC_028135). *Neurospora crassa* (NC_026614) was served as an outgroup. The percentages of replicate trees in which the associated taxa clustered together in the bootstrap test (1000 replicates) are shown next to the branches.

The circus mitogenome was 116,819 bp in length with a GC content of 30.75%, which was one of the most largest mitogenome in Agaricales (135 kbp in *Agaricus bisporus,* Férandon et al. 2013). Gene prediction showed 37 putative protein-coding genes and 23 tRNAs were determined in the genome. However, no large or small rRNA subunits (rnl or rns) were detected in this genome. The 13 conserved protein-coding genes encoded the 6 subunits of NAD dehydrogenase (nad1, nad 3–6 and nad4L genes), 3 cytochrome oxidases (cox1-3), apocytochrome b (cob) and 3 ATP synthases (atp6, apt 8 and apt 9). The set of 23 tRNA genes could code for all 20 standard amino acids. 11 group I introns, 1 group II and 3 unclassified intron were distributed in 4 genes cob (3 introns), cox1 (5 introns), nad1 (5 introns) and nad5 (1 introns).

Phylogenetic relationship based on concatenated protein sequences confirmed that *L. edodes* was clustered together with *Moniliophthora roreri* and *M. perniciosa* belong to the family Marasmiaceae (Matheny et al. 2006), all of which were belong to Agaricales (Figure 1). At the class level, the evolutionarily relationship among Agaricales, Russulales, Polyporales, Cantharellales and Sebacinales was in agreement with the result of our other study on mitogenomes (Yang et al. 2016) (Figure 1). The mitogenome of *L. edodes* would contribute to the understanding of the phylogeny and evolution of Omphalotaceae and Agaricales.

## References

[CIT0001] Chang ST, Li GSF, Peberdy JF. 1985 Isolation of protoplasts from edible fungi. Mircen J Appl Microbiol Biotechnol. 1:185–193.

[CIT0002] Chang ST. 1999 World production of cultivated edible and medicinal mushrooms in 1997 with emphasis on *Lentinus edodes* (Berk.) Sing, in China. Int J Med Mushrooms. 1:291–300.

[CIT0003] Coil D, Jospin G, Darling AE. 2014 A5-miseq: an updated pipeline to assemble microbial genomes from Illumina MiSeq data. Bioinformatics. btu661:1–3. 10.1093/bioinformatics/btu661 25338718

[CIT0004] Férandon C, Xu J, Barroso G. 2013 The 135 kbp mitochondrial genome of *Agaricus bisporus* is the largest known eukaryotic reservoir of group I introns and plasmid-related sequences. Fungal Genet Biol. 55:85–91. 23428625 10.1016/j.fgb.2013.01.009

[CIT0005] Gong WB, Li L, Zhou Y, Bian YB, Kwan HS, Cheung MK, Xiao Y. 2016 Genetic dissection of fruiting body-related traits using quantitative trait loci mapping in *Lentinula edodes*. Appl Microbiol Biotechnol. 100:1–16. 26875873 10.1007/s00253-016-7347-5

[CIT0006] Hibbett DS, Donoghue MJ. 1996 Implications of phylogenetic studies for conservation of genetic diversity in shiitake mushrooms. Conserv Biol. 10:1321–1327.

[CIT0007] Kumar S, Stecher G, Tamura K. 2016 MEGA7: molecular evolutionary genetics analysis version 7.0 for bigger datasets. Mol Biol Evol. doi: 10.1093/molbev/msw054. PMC8210823 27004904

[CIT0008] Li HB, Wu XQ, Peng HZ, Fu LZ, Wei HL, Wu QQ, Jin QY, Li N. 2008 New available SCAR markers: potentially useful in distinguishing a commercial strain of the superior type from other strains of *Lentinula edodes* in China. Appl Microbiol Biotechnol. 81:303–309. 18791710 10.1007/s00253-008-1671-3

[CIT0009] Matheny PB, Curtis JM, Hofstetter V, Aime MC, Moncalvo JM, Ge ZW, Slot JC, Ammirati JF, Baroni TJ, Bougher NL, et al 2006 Major clades of Agaricales: a multilocus phylogenetic overview. Mycologia. 98:982–995. 17486974 10.3852/mycologia.98.6.982

[CIT0010] Thompson BJD, Gibson TJ, Plewniak F, Jeanmougin F, Higgins DG. 2010 The CLUSTAL_X windows interface: exible strategies for multiple sequence alignment aided by quality analysis tool. Nucleic Acids Res. 25:4876–4882. 10.1093/nar/25.24.4876 PMC147148 9396791

[CIT0011] Yang R, Li Y, Li C, Xu J, Bao D. 2016 The complete mitochondrial genome of the Basidiomycete edible fungus *Pleurotus eryngii*. Mitochondrial DNA Part B. 1:772–774. 10.1080/23802359.2016.1238755 PMC7799561 33473623

